# Clinical Significance of Renal Allograft Protocol Biopsies: A Single Tertiary Center Experience in Malaysia

**DOI:** 10.1155/2019/9153875

**Published:** 2019-05-02

**Authors:** Mei Sian Fu, Soo Jin Lim, Maisarah Jalalonmuhali, Kee Seong Ng, Soo Kun Lim, Kok Peng Ng

**Affiliations:** ^1^Department of Medicine, Hospital Segamat, 85000 Segamat, Johor, Malaysia; ^2^Division of Nephrology, Department of Medicine, University Malaya Medical Centre, 59100 Kuala Lumpur, Malaysia; ^3^Department of Medicine, University Malaya Medical Centre, 59100 Kuala Lumpur, Malaysia

## Abstract

**Background:**

The role of protocol renal allograft biopsy in kidney transplantation is controversial due to the concern with procedural-related complications; however, its role is slowly evolving. Recent evidence suggests that protocol biopsy is useful in detecting subclinical renal pathology. Early recognition and treatment of renal pathologies can improve long-term outcomes of renal allografts.

**Methodology:**

A total of 362 renal allograft protocol biopsies were performed in adult recipients of kidney transplantation between 2012 and 2017. After excluding those with poor quality or those performed with a baseline serum creatinine level >200 umol/L, we analyzed 334 (92.3%) biopsies. Histology reports were reviewed and categorized into histoimmunological and nonimmunological changes. The immunological changes were subcategorized into the following: (1) no acute rejection (NR), (2) borderline changes (BC), and (3) subclinical rejection (SCR). Nonimmunological changes were subcategorized into the following: (1) chronicity including interstitial fibrosis/tubular atrophy (IFTA), chronic T-cell-mediated rejection (TCMR), unspecified chronic lesions, and arterionephrosclerosis, (2) de novo glomerulopathy/recurrence of primary disease (RP), and (3) other clinically unsuspected lesions (acute pyelonephritis, calcineurin inhibitors toxicity, postinfective glomerulonephritis, and BK virus nephropathy). Risk factors associated with SCR were assessed.

**Results:**

For the histoimmunological changes, 161 (48.2%) showed NR, 145 (43.4%) were BC, and 28 (8.4%) were SCR. These clinical events were more pronounced for the first 5 years; our data showed BC accounted for 59 (36.4%), 64 (54.2%), and 22 (40.7%) biopsies within <1 year, 1-5 years, and > 5 years, respectively (p = 0.011). Meanwhile, the incidence for SCR was 6 (3.7%) biopsies in <1 year, 18 (15.3%) in 1-5 years, and 4 (7.4%) in >5 years after transplantation (p=0.003). For the nonimmunological changes, chronicity, de novo glomerulopathy/RP, and other clinically unsuspected lesions were seen in 40 (12%), 10 (3%), and 12 (3.6%) biopsies, respectively. Living-related donor recipients were associated with decreased SCR (p=0.007).

**Conclusions:**

Despite having a stable renal function, our transplant recipients had a significant number of subclinical rejection on renal allograft biopsies.

## 1. Introduction

Renal biopsy is the gold standard in determining the cause of renal allograft dysfunction. Renal allograft protocol biopsy is defined as biopsy performed at predefined intervals after transplantation, which is unrelated to graft dysfunction. Traditionally, the indications of renal allograft biopsy were either due to the changes in the patient's clinical condition or abnormal renal biochemical parameters.

For the past few decades, there has been a paradigm shift in the indications of renal allograft biopsies. Several studies suggested that early acute rejection episodes and chronic changes in the allograft kidneys were often subclinical without a concomitant rise in serum creatinine or proteinuria [1–4]. Hence, performing a preemptive renal allograft biopsy may help with identification of acute or chronic rejection as it may potentially alter the outcome of renal allograft that is amenable to treatment.

Due to the above findings, some centers have started to implement protocol biopsy program. Acceptance of protocol biopsy is gaining momentum worldwide in view of recent studies which suggest that protocol biopsy is useful in detecting subclinical rejection (SCR), defined as histopathological evidence of acute tubulitis in the presence of stable kidney function [5–8]. Early recognition and treatment of SCR may improve long-term renal outcomes [9, 10].

In contrast to laboratory values, protocol biopsies can track chronic histologic changes in different compartments of the allograft, providing a more detailed picture of the allograft health. Protocol biopsies can also reveal unsuspected findings and influence therapy in the majority of patients. Other potentially reversible chronic pathologies such as chronic T-cell or antibody-mediated rejection, de novo glomerulopathy or recurrent disease, BK virus nephropathy, interstitial fibrosis and tubular atrophy (IFTA), and calcineurin-inhibitor nephrotoxicity may be detected, which allow modification of therapy to limit ongoing graft injury [11–15].

In this study, we examined the usefulness of protocol biopsy in detecting subclinical rejection and other unsuspected lesions in patients with stable graft function and assessed the risk factors that may influence SCR.

## 2. Materials and Methodology

This is a retrospective observational study. All adult kidney transplant recipients with at least one protocol biopsy performed at the University Malaya of Medical Center (UMMC) between January 2012 and June 2017 were eligible. We recruited all adult recipients of either living or cadaveric renal transplant with a variability of serum creatinine of less than 15% from baseline [16–18], and there was no change in immunosuppressive regimen from the last follow-up till the biopsy date.

We excluded biopsies with a baseline serum creatinine level >200 umol/L or poor quality of renal biopsy specimens (e.g., absence of renal tissues, inappropriate fixing).

The total number of protocol biopsies performed between January 2012 and June 2016 was 362, of which 334 biopsies were analyzed in this study. We excluded 23 biopsies with inadequate tissues and 5 biopsies which were performed in patients with baseline serum creatinine level >200 umol/L. An adequate biopsy was defined as a specimen able to be interpreted by the histopathologist.

The biopsy was done by a nephrologist using a 16G biopsy needle under ultrasound guidance. After the procedure, the patient was confined to bed rest for 6 hours with blood pressure and urine monitoring. Patients who were asymptomatic and hemodynamically stable were discharged on the same day. All biopsies' reports were reviewed. Based on the Banff 2007 Classification [19, 20], we categorized the histological findings into NR, with BC and SCR. In addition, we evaluated the biopsy according to nonimmunological changes, i.e., CNI-induced nephropathy, de novo glomerulopathy or RP, IFTA, postinfection GN, BK nephropathy, arterionephrosclerosis, and acute pyelonephritis.

We retrieved all data from both the electronic and paper-based medical records, which included the demographic characteristics (such as age and gender of recipients and donors), causes of ESRD, types of donors (related vs. unrelated), the number of HLA-DR antigen mismatches, and the type of immunosuppressants used. Data collected were analyzed using SPSS version 23.

Age, HLA, serum Cr were expressed by mean ± SD. Chi-square or Fisher exact test was used for between-group comparisons of categorical variables. One-way ANOVA and Kruskal–Wallis test were used for between-group comparisons of continuous variables.* P*<0.05 was considered significant. Data were analyzed using the software package SPSS for Windows release 23 (SPSS, Inc., USA).

## 3. Results

### 3.1. The Histological Finding of Protocol Biopsy in Renal Transplant Recipients

**Figure 1:**
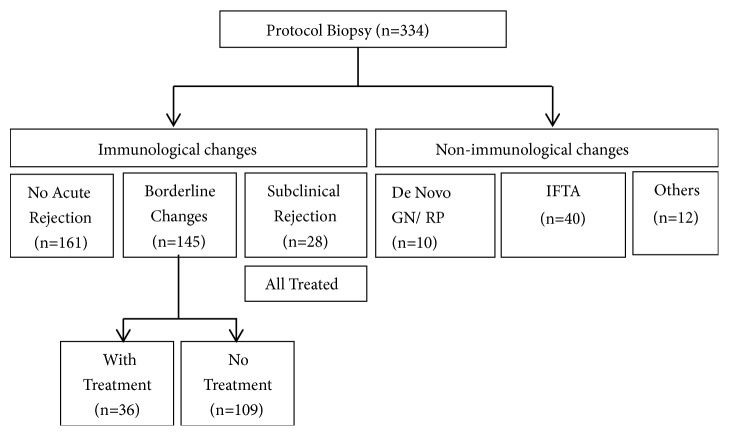
Overview of renal protocol biopsies.

A total of 334 biopsy specimens from 147 renal allograft recipients were evaluated. The timing of biopsies ranged from one month to 22.8 years after transplantation. Each transplant recipient underwent between one and seven separate biopsies. Biopsy outcome was subsequently divided based on either histoimmunological or nonimmunological changes as illustrated in Figure 1.

### 3.2. Analysis of Protocol Biopsy according to Histoimmunological Changes

For histoimmunopathologic forms of acute rejection, according to the Banff criteria, 161 biopsies (48.2%), 145 biopsies (43.4%), and 28 biopsies (8.4%) were reported as NR, BC, and SCR, respectively. For the subgroups of SCR, we detected Type 1A in 17 biopsies, Type 1B in 10 biopsies, and Type IIA in one biopsy.

We subdivided the biopsies according the timing of the biopsies performed after transplantation to < 1 year (n= 162), 1-5 years (n=118), and > 5 years of transplantation (n=54). In BC group, 59 biopsies (36.4%) were detected during first year of transplantation, 64 biopsies (54.2 %) during 1-5 years after transplantation, and 22 biopsies (40.7%) after 5 years of transplantation; p value = 0.011 (refer to Table 2). Among the SCR group, 6 biopsies (3.7%) were detected during < 1 year after transplantation, 18 biopsies (15.3%) within 1-5 years after transplantation, and 4 biopsies (7.4%) after 5 years of transplantation; p value = 0.003 (refer to Table 2).

Table 1 shows a comparison of the clinical characteristics of each group prior to the biopsy. There were no differences between the recipients in age, sex, mean serum creatinine, and eGFR level at the time of the protocol biopsy. The causes of primary disease were not significantly different between the groups.

### 3.3. Risk Factors That Influence Subclinical Rejection

#### 3.3.1. Donor Status

**Figure 2:**
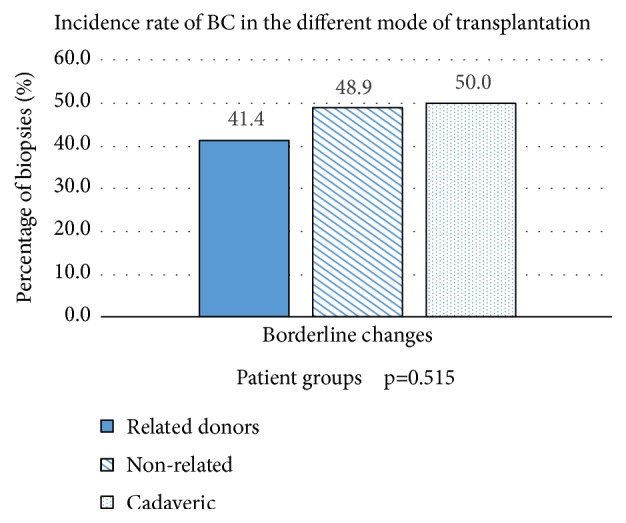
Incidence rate of BC in the different mode of transplantation.

**Figure 3:**
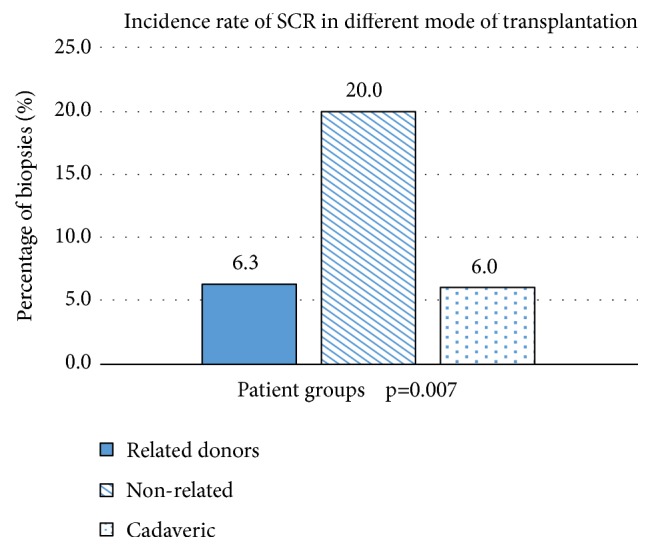
Incidence rate of SCR in different mode of transplantation.

Donor source can be divided into related versus unrelated living and cadaveric. Among 334 renal allograft biopsies, 237 biopsies were retrieved from living-related donors, 45 from living-unrelated donors, 50 biopsies from cadaveric donors, and two from unknown donors. The proportion of NR or BC was not significantly different between living-related, living-unrelated, and cadaveric donors (NR, 52.3% vs. 31.1% vs. 44%, p=0.06; BC, 41.4% vs. 48.9% vs. 50%, p = 0.515, respectively; Figure 2). However, the proportion of SCR was lower in living-related, cadaveric donors, and living-unrelated donors (6.3% vs. 6% vs. 20%; p = 0.007; Figure 3).

#### 3.3.2. HLA-DR Antigen Mismatches

**Figure 4:**
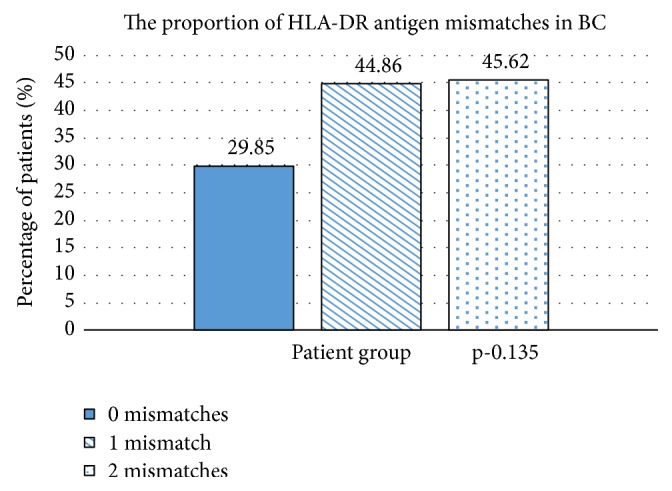
The proportion of HLA-DR antigen mismatches in BC.

**Figure 5:**
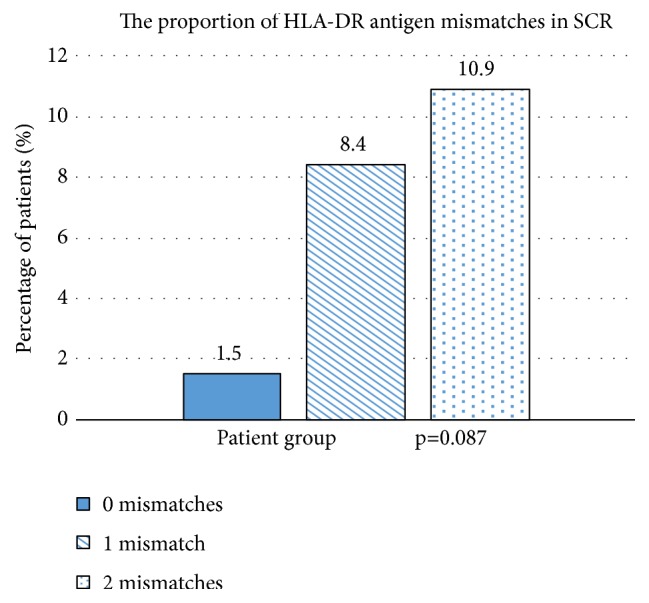
The proportion of HLA-DR antigen mismatches in SCR.

The total number of HLA-DR antigen mismatches can influence the histological findings. Compared with NR and BC, we reported a higher degree of HLA-DR antigen mismatches in SCR. In our study, for the effects of HLA-DR antigen mismatches, we only look at the living-related donor's biopsies (n=220). In BC group, 29.85% of biopsies have no HLA-DR antigen mismatches, 44.86% have one, and 45.62% have two (Figure 4). In SCR group, only 1.5% of biopsies have no HLA-DR antigen mismatches, but one HLA-DR antigen mismatch significantly increased the proportion to 8.4%, and an even higher increase in the SCR rate was observed in patients with two HLA-DR antigen mismatches (10.9%; Figure 5).

#### 3.3.3. Types of Immunosuppressants

The majority of transplant recipients, treated with either cyclosporine A (CsA) or tacrolimus (TAC), showed no significant between-group difference in histological findings (NR, 51.9% vs. 48.8%; BC, 41.5% vs. 43.4%, p = 0.711; SCR, 6.6% vs. 7.9%, p = 0.468).

### 3.4. Management of Patients after Protocol Biopsies

All patients with SCR were treated with three daily intravenous pulses of 500 mg of methylprednisolone.

For BC, only 36 patients were being treated with the same regimen as SCR, the remaining 109 BC patients did not receive any specific treatment based on clinician judgment.

For patients with NR, we followed them up as per usual protocol. All recipients received a triple-drug immunosuppressive regime consisting of a CNI (CsA or TAC) or mTOR inhibitors (sirolimus or everolimus), an antimetabolite (mycophenolic acid or azathioprine), and prednisolone.

### 3.5. The Incidence of Nonimmunological Changes in a Protocol Biopsy

Of 334 protocol biopsies performed, chronicity was observed in 40 (12%) biopsies, with subclassifications as follows: 27 interstitial fibrosis/tubular atrophy (IFTA), five TCMR, six unspecified chronic lesions, and two arterionephrosclerosis. De novo glomerulopathy or RP was seen in 10 (3%) biopsies. Meanwhile, 12 (3.6%) of biopsies showed other nonimmunological changes, such as acute pyelonephritis (5/334), CNI toxicity (4/334), postinfective GN (1/334), and BK virus nephropathy (2/334).

## 4. Discussions

Protocol biopsies are not routinely performed universally in view of their marginal utility and potential risks. The largest retrospective audit of a total of 2,127 adult allograft renal biopsies was assessed for major complications, and 1,486 were assessed for minor ones in four major transplant centers in Europe [21]. Another prospective study, done in 2005 with 1,171 protocol biopsies performed in 508 patients at 6, 12, and 26 weeks after transplantation, had also shown that the benefits of protocol biopsy outweigh the risks with acceptable complications [22].

Since 2009, our center has been performing biopsies in recipients with stable graft function. In 2015, we extended the service by performing biopsy routinely at 1, 3, 6, and 12 months after transplantation, followed by annual biopsy for monitoring. In our analysis, by performing biopsies in patients with normal stable graft function, we are able to detect incident BC, SCR, and unsuspected lesions (e.g., chronicity, CNI toxicity, RP or de novo glomerulopathy, BK virus nephropathy, and asymptomatic urinary tract infections). This early detection can help to improve the long-term graft survival [15].

### 4.1. Detection of SCR and BC

We found 43.4% BC and 8.4% SCR. On the one hand, these results were similar to a study conducted in Korea. This Korean study, reporting a 10-year experience of protocol biopsy performed at day 14 after transplantation in 304 living-related renal transplant recipients with stable graft function, found 37.8% BC and 13.2% SCR incidence [6]. On the other hand, these results were much more different compared with other previous results with biopsies performed in different periods after transplantation [2, 4, 13, 17, 23, 24]. In the latest published study in 2015, they reported 84.6% NR and 10.7% SCR in a 13-year retrospective study which enrolled 174 adult renal transplant recipients with a protocol biopsy performed at 30 days after transplantation [25]. Our subdivision group, according to the timing of biopsy posttransplantation, also noted that more SCR incidences were detected if we performed biopsy many years after transplantation; hence we recommended that early protocol biopsy is needed for early detection of pathology. We reported a slightly different results compared to other studies due to the retrospective nature of our study, different timings of the biopsies, different inclusion criteria, and other factors such as immunosuppression, donor source, and numbers of HLA-mismatches.

The use of immunosuppression is one of the important factors that can affect the incidence of SCR. Nankivell et al. [2] reported that CsA-treated patients had a higher risk of SCR, compared with the TAC-treated group. They also reported that the use of TAC or combination of TAC and MMF reduced the incidence of SCR. Gloor et al. [26] reported 2.6% SCR at 3 months after transplantation in patients on TAC and MMF. In our study, we did not observe any difference between TAC and CsA groups, which was similar to the result reported by Choi et al. [6]. This may be due to different timings of biopsies and the effects of TAC on cadaveric renal transplantation not well reported.

Choi et al. [6] reported that HLA-DR mismatch and unrelated donor status were associated with higher risk of SCR. They found a positive dose-response relationship between the incidence of SCR and the number of HLA-DR mismatches. In patients without HLA-DR mismatch, 2.7% of them developed SCR, while the incidence rates were 15.4% and 20.8% in patients with one and two HLA-DR mismatches, respectively. Rush et al. [27] reported that 20% of patients without HLA-DR mismatch developed SCR, while the incidence rates were 30% and 63% in patients with one and two HLA-DR mismatches, respectively, in a 1-month protocol biopsy. The corresponding rates in our study were 1.5%, 8.4%, and 10.9%. This finding may suggest that the HLA-DR matched kidney has less early rejection compared to those with more mismatches.

The number of living-unrelated renal transplants performed has reduced over the years due to the reduction in commercial transplantation. In our study, we reported a higher proportion of SCR in living-unrelated than living-related renal transplantation (6.3% vs. 20%), which was similar to Choi et al. (10.1% vs. 19%) and Fuller et al. (18.5% vs. 30%) [6, 28]. Therefore, early and intensive immunosuppression may be needed in those living-unrelated recipients to prevent early rejection [6]. We also revealed that the rate of SCR in our cadaveric was 6.0%, which was lower than other studies [4, 23]. As we selected those low immunological risk recipients for cadaveric transplantation, our smaller sample size than other studies may have contributed to this difference.

The early detection and treatment of SCR may benefit from graft outcome and the histological findings [29]. In our study, we treated all patients with SCR using pulsed intravenous (IV) methylprednisolone for three days. During follow-up, we noticed that one of them developed graft failure after 18 weeks of SCR detection. Rush et al. [16] reported that SCR treatment in the early posttransplantation decreased chronic tubulointerstitial score and late clinical rejection episodes, leading to improvement in the long-term graft survival and a lower serum creatinine at 24 months, compared with control patients who did not receive pulsed IV methylprednisolone. In addition, Nankivell et al. and Legendre et al. showed an increase in IF/TA in subsequent biopsies in untreated SCR group [1, 2, 24]. Choi et al. demonstrated a decrease in 10-year graft survival rate with untreated SCR (obtained from biopsy at two weeks after transplantation), compared to normal histological findings

### 4.2. Detection of Nonimmunological Changes (Clinical Unsuspected Lesions)

Rush et al. [30] reported that protocol biopsy is useful for the detection of unexpected pathology in recipients with good functioning grafts, e.g., early chronic changes (IF/TA) and transplant glomerulopathy (TG). Chronic TCMR remains poorly defined. CAN was used in many studies, but in view of its nonspecificity, CAN is no longer part of the vocabulary of transplant pathology. In our study, we classified the chronicity into IFTA, chronic rejection (CR), and arterionephrosclerosis, based on the histopathological examination reports.

The incidence of CAN or IFTA has been reported in several studies. Nankivell et al. reported a prevalence of CAN from 24% up to 40%, while Seron et al. reported a prevalence of CAN in about 42% of protocol biopsies at 3 months after transplantation [2, 17]. Fujisawa et al. found CAN in 30.4% of paediatric recipients at approximately 100 days after living-related renal transplantation [31]. Legendre et al. reported the prevalence of IF/TA as 25% at 3 months and 50% at 2 years in 41 patients with normal graft function [1, 24]. In our study, we reported 12% of chronicity due to our timing of biopsy which ranged from 2 weeks to 22.8 years after transplantation. Further studies will be required to determine the incidence of IFTA in our center.

Briganti et al. and Ivanyi B reported that glomerular disease can either recur or appear de novo in transplant recipients. It contributes to 8.4% graft failures by 10 years [32, 33]. We found that de novo glomerulopathy or RP occurred in 3% of patients in our study.

CNI is the standard of care for immunosuppression after renal transplantation but the potential nephrotoxicity has been reported [34, 35]. Nankivell reported that both CsA and TAC produced similar fibrogenic effects and pattern of nephrotoxicity in the kidney [2]. Takeda et al. reported an incidence of CsA nephrotoxicity in up to 42% of patients who had protocol biopsies performed at 12 months after transplantation [36]. The reported frequency of CsA nephrotoxicity varied from 10 to 54% in renal transplantation [37, 38]. Legendre et al. found a higher trough level of CsA in patients with histological deterioration than in those within the range of 2-year posttransplantation protocol biopsies [1]. We found only 1.2% CNI toxicity which was all within trough level limit. We adjusted and tapered down the dose for CNI after reviewing the results.

BK virus nephropathy has a reported incidence of 1-10% [14] but can result in up to 45% graft loss in affected recipients [39]. Therefore, early diagnosis is needed to resolve the infection and prevent chronic changes. In our study, we detected two biopsies with BK virus nephropathy and managed by dose reduction of immunosuppressants.

Even though we have managed to identify and treat some of our patients with BC and SCR early, there is still a room for improvement of doing single antigen bead Luminex assay at the time of biopsy. This could perhaps identify some of the high risk groups in developing transplant glomerulopathy.

## 5. Conclusion

This study showed that introduction of protocol biopsies in renal transplant allograft recipients has undoubtedly improved patient's management in detecting SCR, BC, and clinically unsuspected lesions. Timely treatment of allograft rejection that cannot be diagnosed on clinical grounds is definitely of benefit to long-term graft function.

Protocol biopsies may also help to monitor the effectiveness of immunosuppressive regimens and inform about the safety of reducing overall immunosuppression in the presence of normal histology.

In addition, protocol biopsies are useful for differentiating chronic loss of renal function caused by immunologic causes from that caused by nonimmunological causes. Based on our study, protocol biopsy is safe with acceptable complication rates, which should be routinely performed for optimal patient care.

## Figures and Tables

**Table 1:** Clinical characteristic of transplant recipients, stratified by type of histological findings.

	*N (n= 161)*	*BC (n= 145)*	*SCR (n= 28)*	p value
Recipient age	42.00 (±11.1)	42.57 (±10.9)	42.71 (±12.5)	0.89
Recipient sex (M:F)	2.83 : 1	2.63 : 1	2.11 : 1	0.796
Cause of ESRD				
DM (n=39)	41%	53.80%	5.20%	0.643
HTN (n=31)	54.80%	38.70%	6.50%	0.16
CGN (n=100)	55%	40%	5%	0.31
Obstructive uropathy (n=16)	56.30%	31.30%	12.40%	0.784
APKD (n=10)	50%	40%	10%	0.967
Analgesic nephropathy (n=2)	0%	100%	0%	0.269
Unknown cause (n=143)	46.20%	42.70%	11.10%	0.226
Serum Creatinine (mmol/L)	110.25 (±26.6)	111.81 (±30.14)	109.6 (±31.86)	0.944
eGFR mL/min1.73 m^2^	67.80 (±15.35)	67.11 (±16.25)	71.28 (±13.58)	0.842

ESRD: end-stage renal disease.

DM: diabetes mellitus.

HTN: hypertension.

CGN: chronic glomerulonephritis.

APKD: adult polycystic kidney disease.

eGFR: estimated glomerular filtration rate.

**Table 2:** The number of the biopsies and the incidence rate according to the timing of biopsy.

Immunopathological form	The number of the biopsies and the incidence rate according to the timing of biopsy			
	< 1 year	1- 5 years	> 5 years	P value
BC	59 (36.4%)	64 (54.2%)	22 (40.7%)	0.011
SCR	6 (3.7%)	18 (15.3%)	4 (7.4%)	0.003

## Data Availability

The data used to support the findings of this study are included within the article.

## Abbreviations

APKD: Adult polycystic kidney disease
BC: Borderline changes
CGN: Chronic glomerulonephritis
eGFR: Estimated glomerular filtration rate
ESRD: End stage renal disease
HTN: Hypertension
IFTA: Interstitial fibrosis/tubular atrophy
NR: No acute rejection
RP: Recurrence of primary disease
SCR: Subclinical rejection
TCMR: T-cell-mediated rejection.
